# A bibliometric analysis of global research trends of inflammation in cervical cancer: A review

**DOI:** 10.1097/MD.0000000000036598

**Published:** 2023-12-08

**Authors:** Meili Kang, Junling Qiu, Hong Wei, Jianing Li

**Affiliations:** a Central Laboratory of Medicine School, Shaanxi Province University Engineering Research Center of Biosecurity Defense Equipment, Xi’an Peihua University, Xi’an, China; b Department of cardiology, First Hospital of Northwestern University, Xi’an, Shaanxi, China; c Department of Rehabilitation Teaching and Research, Xi’an Siyuan University, Xi’an, China; d Department of Medicine School, Xiamen University, Xiamen, China; e Department of Obstetrics and Gynecology, Clinical Medical Research Center for Obstetrics and Gynecology Diseases of Fujian Province, Laboratory of Research and Diagnosis of Gynecological Diseases of Xiamen City, The First Affiliated Hospital of Xiamen University, Xiamen, Fujian, China.

**Keywords:** bibliometric analysis, cell death, cervical cancer, inflammation, Web of Science

## Abstract

Cervical cancer is a common malignant tumor and a leading cause of death in women worldwide. It plays a crucial role in tumorigenesis and progression of cervical cancer. A total of 1606 references on inflammation in cervical cancer were retrieved from the Web of Science Core Collection and visual analysis was performed using VOSviewer. Inflammation in cervical cancer has attracted the attention of researchers. Even though China is the country that publishes the most papers, with the most of the top-ranking institutions, there is no extensive collaboration and exchange of papers by Chinese scholars. *PLOS One* is a popular journal on inflammation in cervical cancer. Instead, authors from other countries perform better, for example, the Sjoerd H. Van Der Burg is the most widely cited author and “M2 macrophages induced by prostaglandin E2 and IL-6 from cervical carcinoma are switched to activated M1 macrophages by CD4 + Th1 cells” (Moniek Heusinkveld, Leiden University Medical Center) is the most cited article of inflammation in cervical cancer. Keywords associated with “apoptosis,” “HPV,” “NF-κB,” and “oxidative stress have been used in many studies, and keywords associated with “apoptosis,” “human papillomavirus (HPV),” “NF-κB,” and “oxidative stress” are involved in many studies, and there may be more research ideas in the future. From the perspective of precision medicine, more substantive research articles can promote scientific value, strengthen communication and cooperation, produce more extensive research results, and greatly promote the clinical diagnosis and treatment of cervical cancer. All procedures performed in this study involving human participants were in accordance with the ethical standards of the institutional and/or national research committee and with the 1964 Helsinki Declaration and its later amendments or comparable ethical standards.

## 1. Introduction

Cervical cancer is a common malignant tumor and a leading cause of death among women worldwide.^[1]^ According to the Global Cancer Statistics 2020, there were an annual incidence around 604,127 cases and 341,831 deaths from cervical cancer worldwide in 2020, which accounted 6.5% and 7.7% of all cancer cases and deaths among women, respectively.^[2]^ And more than 85% of cervical cancer cases and 90% of deaths occur in developing countries.^[3]^ Cervical cancer is preventable; however, because of the lack of cervical cancer screening and human papillomavirus (HPV) vaccines in developing countries, it has a huge global and public health impact.^[3,4]^ Although the world first HPV vaccine became available in 2006 and governments invested heavily in cervical cancer screening, screening systems still need to be significantly improved in terms of health service capacity, optimizing screening strategies, and prioritizing financial investments and commitment to health.^[5]^ Thus, for most low-income countries, the goal of eliminating cervical cancer seems to be close but out of reach.

The clinical use of HPV vaccines and screening is based on the fact that persistent infection with high-risk HPV types is a major risk factor for cervical cancer. Oncogenic proteins encoded by HPV E6/E7 genes are important factors leading to cervical epithelial carcinogenesis.^[6,7]^ The binding and regulation of these 2 proteins by p53 and pRb, 2 major intracellular tumor suppressor proteins, can significantly alter the cell growth cycle, DNA repair, and activation of the cyclooxygenase-2 inflammatory pathway, resulting in increased inflammation and tumorigenesis.^[8,9]^ Therefore, inflammation plays a crucial role in the tumorigenesis of cervical cancer.

Studies have confirmed that the tumor microenvironment of cervical cancer is associated with the production of several cytokines, including pro-inflammatory cyclooxygenase-2, tumor necrosis factor-alpha, interleukin 1 (IL-1), IL-6, IL-17, IL-18, hypoxia-inducible factor-1, reactive oxygen species, and anti-inflammatory factors IL-10 and transforming growth factor beta; thus, changes in these components can be observed throughout the pathological process.^[8,10]^ Tumor necrosis factor-alpha triggers other inflammatory mediators that participate in inflammatory responses and promote tumorigenesis. In cervical cells, it induces bidirectional regulatory proteins that, together with IL-1α, stimulate the proliferation of permanent cervical cells.^[11]^ IL-6 regulates the vascular endothelial growth factor-dependent malignancy of cervical cancer cells by activating the Janus kinases/signal transducer and activator of transcription 3 (STAT3) and rat sarcoma/mitogen-activated protein kinases signaling pathways.^[12]^ Serum IL-6 levels can be used as diagnostic and prognostic indicators for cervical cancer.^[13]^ IL-6 and IL-10 have been implicated in the development of HPV-induced cervical lesions and inhibition of immune cells, which are important for viral elimination.^[14]^ The immunoregulatory cytokine IL-10 decreases the expression of histocompatibility complex class I molecules in cervical cancer cells^[10,15]^ and protects against the cytotoxic activity of cytotoxic T lymphocytes.^[15]^ purinergic ligand-gated ion channel 7 and adenosine A(2A) receptors are considered novel therapeutic targets because blocking purinergic ligand-gated ion channel 7 results in reduced release of proinflammatory cytokines, whereas blocking adenosine A(2A) increases the activation of cytotoxic T lymphocytes to fight cervical cancer.^[8]^ Increasing dietary intake of natural anti-inflammatory compounds such as curcumin and resveratrol helps prevent the development of cancer.^[16,17]^ Anti-inflammatory drugs typically target oncogenic viruses to generate signaling pathways for persistent infection, thereby countering the role of persistent infection in cancer progression.^[18]^ Therefore, a more accurate description of the potential role of cancer-related inflammatory biomarkers would be of great help in the diagnosis and treatment of cervical cancer.

In this study, we performed a bibliometric analysis to gather a diagrammatic drawing of inflammation in cervical cancer, which can evaluate the current status and trends of research and predict the research prospects of a given topic.^[19,20]^

## 2. Methods

### 2.1. Study selection

We retrieved all literature data regarding inflammation in cervical cancer indexed in the Web of Science (WOS) core collection. The articles from 2000 to 2022 (December 31, 2021) were searched and the search terms are as follow: # 1, “cervical cancer” OR “cervix cancer” OR “cervix neoplasm” OR “cervical neoplasm”; # 2, “inflammation” OR “inflammatory response” OR “inflammatory cytokine”; # 3, “# 1” AND “# 2”; #4, #3 AND “English” AND “Article and Review.”

### 2.2. Data collection and statistical analysis

A total of 1606 documents from #4 were retrieved from the WOS database, and visual analysis was performed using VOSviewer according to previous studies.^[19,21]^ The title, publication year, authors, country, institution, keywords, journal, citation frequency, and relative citation ratio (obtained from the National Institutes of Health [NIH] Open Citation Collection: https://icite.od.nih.gov/analysis) were obtained for each article. The 2021 impact factor (IF) of the journals was obtained from Journal Citation Reports on May 1, 2023.

To extract the most common topics, impactful authors, and institutions, we chose the keywords and key references, and the visualization of collaboration networks was conducted using VOSviewer version 1.6.18 (Leiden University, Leiden, Netherlands). We chose keywords and key references to predict research prospects and hotspots. Keywords and key references were analyzed using VOSviewer and CiteSpace, respectively. The parameters of VOSviewer were set as follows: method (linlog/modularity).

## 3. Results

### 3.1. Publication outputs

**Figure 1.**
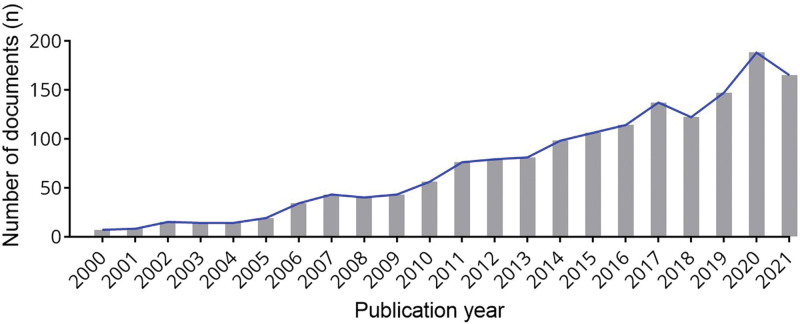
Annual number of documents indexed in the WOS and WOSCC from 2000 to 2021 by the online bibliometric analysis. WOS = Web of Science.

1606 documents of English Articles and Reviews were retrieved from the WOS Core Collection database. The change in the output of inflammation in cervical cancer is increasing (Fig. 1). In recent years, it has gradually achieved a high yield, that is, it has attracted wide attention and interest. As of August 31, 2023, there were 200 articles, and research results will continue to increase in the later period.

### 3.2. Funding and database

A total of 1151 funding sources supported research on inflammation in cervical cancer. The top ten major sources of funding are listed in Table S1, http://links.lww.com/MD/L37. The Natural Science Foundation of China is the main source of funding in China, while the NIH is the main source of funding in the United States. The National Natural Science Foundation of China (n = 122), United States Department of Health Human Services (n = 104), NIH USA (n = 103), NIH National Cancer Institute NCI (n = 68), and NIH National Institute of Allergy Infectious Diseases NIAID (n = 23) were the top 5 funding sources.

### 3.3. Countries/regions and organization

As shown in Table S2, http://links.lww.com/MD/L38, the United States of America (USA n = 194) and China (n = 336) are the top 2 published papers, followed by India, Japan, Brazil, Germany, South Korea, Italy, the UK, and Iran. The citations of the USA (n = 6367) and China (n = 5761) were the most common, but the USA had the highest total link strength and links (Table S2, http://links.lww.com/MD/L38), suggesting that American articles are more influential.

**Figure 2.**
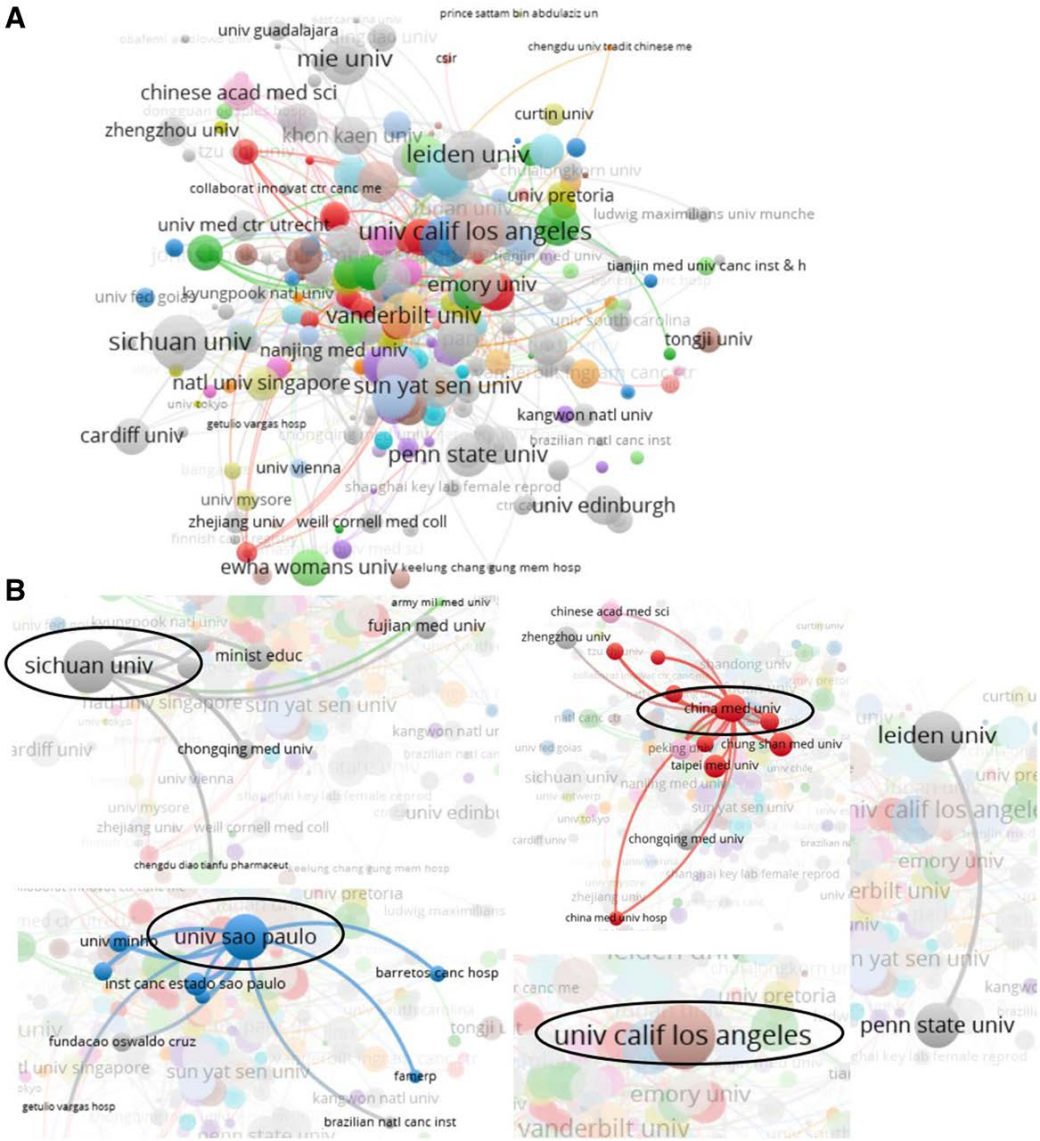
Coauthor analysis of organizations with network visualization. (A) The largest set of 816 valid items (1656 total items) were interlinked. (B) Some partners of organizations were performed.

Table S3, http://links.lww.com/MD/L39 shows the top ten institutions in terms of publications, which mainly come from China, the USA, Brazil, and Mexico, with 6 seats in Chinese institutions. Sichuan University (n = 22, China), the University of California (n = 18, USA), and the University of São Paulo (n = 17, Brazil) were the top 3 publications and citations. The University of California in the United States had a clear preference for citations (896 citations), and China Medical University had the highest total link strength (n = 44) (Table S3, http://links.lww.com/MD/L39). According to the statistical analysis, some of the publications were completed in cooperation with multiple institutions, and each top organization had relationships with other institutions (Fig. 2). Some of the 1656 items in the network were not connected, and the largest set of connected items consisted of 816 items (Fig. 2A). Some institutions stand alone; for example, Tongji University is an isolated organization (shown by the black arrow in Fig. 2A) that is not aligned with other outside institutions. Sichuan University, University of São Paulo, and China Medicinal University also keep in touch with several partners, but their partners look weak. The University of California, Los Angeles, has no contact with relevant agencies, and Leiden University (Netherlands) and Penn State University (USA) are important partners for each other and have similar academic influence (Fig. 2B). The data suggest that these top institutions lack cooperation and communication with each other and that Chinese institutions should improve cooperation with their counterparts in other countries.

### 3.4. Journals analysis

A total of 541 journals have published research documents related to inflammation in cervical cancer. As shown in Table S4http://links.lww.com/MD/L40, the top ten journals included 29.76% of production (161/541). *PLOS One* is the most dynamic journal on inflammation in cervical cancer, followed by *Oncology Letters, International Journal of Gynecological Cancer, Oncotarget, Scientific Reports, International Journal of Clinical and Experimental Pathology, Oncology Reports, Asian Pacific Journal of Cancer Prevention, Mediators of Inflammation* and *Gynecologic Oncology*. The IF of the 10 journals ranged from 3.111 to 5.304, and *Gynecologic Oncology* showed a maximum IF of 5.304 (Q1). *Oncotarget, International Journal of Clinical and Experimental Pathology*, and *Asian Pacific Journal of Cancer Prevention* were without IF because they were not included in the latest JCR Journal citation report (Table S4, http://links.lww.com/MD/L40). There are also Top journals in this field such as *JAMA Neurology, Nature Communications, Nature Reviews Microbiology, Nature Reviews Urology*. Therefore, *PLOS One* is a popular journal on inflammation in cervical cancer. However, when choosing a journal, we should avoid journals with low reputations and make careful decisions.

### 3.5. Authors analysis

**Figure 3.**
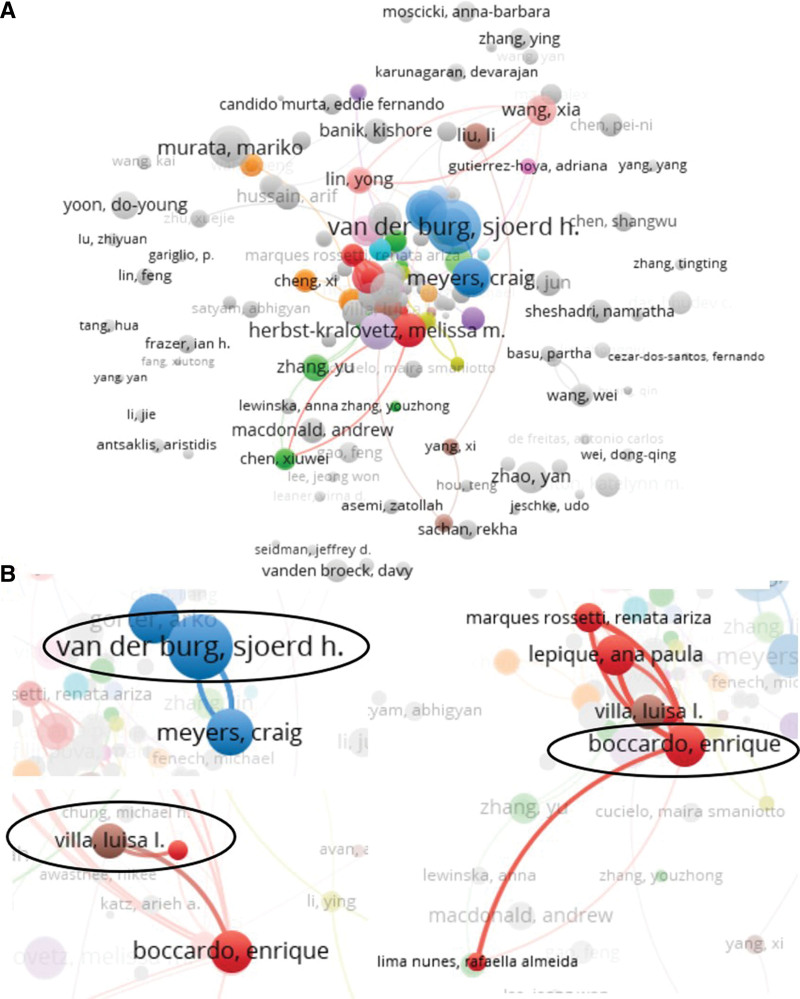
Co-occurrence analysis of authors. (A) 476 valid items (6573 total items) were connected. (B) The cooperation of Van Der Burg, Luisa L. Villa, and Enrique Boccardo were presented.

A total of 6573 authors have been devoted to studying inflammation in cervical cancer. Luisa L. Villa is the most active author in this field (11 documents and 331 citations), followed by Enrique Boccardo, Ana P. Lepique, Sjoerd H. Van Der Burg (Leiden University Medical Center, Netherlands), and Yu Zhang (Harbin Medical University, China) are close behind. The fifth and seventh places are Christoph Grimm, Stephan Polterauer, and Alexander Reinthaller from Christoph Grimm (Austria) with 5 documents and 139 citations. Melissa M. Herbst-Kralovetz and Pawel Laniewski are from the University of Arizona (USA) and are ranked 9th and 10th, respectively (with 5 documents and 261 citations). However, Sjoerd H. Van Der Burg documents were the most widely cited of these authors, with 6 documents and 626 citations (Table S5, http://links.lww.com/MD/L41). The co-authorship map of the authors was generated using VOSviewer (Fig. 3), and 476 valid items (6573 total items) were connected (Fig. 3A). Some researchers are also distributed independently of other active scholars, and the connections between authors are not strong. Although Sjoerd H. Van Der Burg and Luisa L. Villa are prolific and widely influential scholars in their fields, their interaction with other scholars is weak, and Enrique Boccardo is a relatively active author (Fig. 3B). The data show that most collaborations between authors are limited to their own teams and institutions, and there is still a lack of external communication and cooperation.

### 3.6. Citation analysis

**Figure 4.**
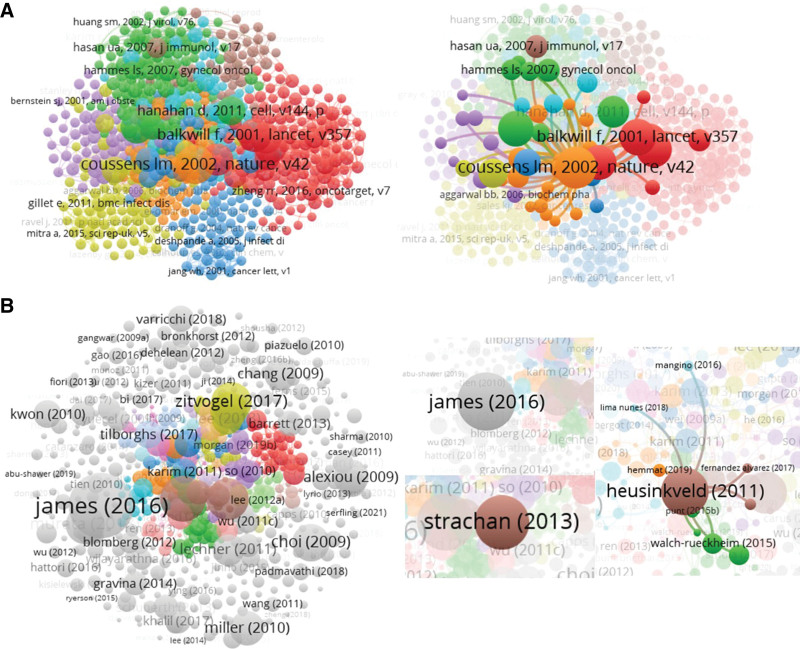
Citation analysis of documents. (A) Of the 45,921 cited reference, 572 meet the threshold (minimum number of documents of an author: 5) and the co-citation map of cited references was generated. (B) Of the 1069 documents, 572 meet the threshold (minimum number of citations of a document: 10) and the citation map of documents was generated.

“Inflammation and cancer” (Nature, 2022) is the highest cited reference of inflammation in cervical cancer (with 88 citations) (Table S6, http://links.lww.com/MD/L42), and it also becomes the core of the other cited references and liaises extensively with them (Fig. 4A). The front rankings were Lisa M Coussens (Nature, 2002), Fran Balkwill (Lancet, 2001), Alberto Mantovani (Nature, 2008), Jan MM Walboomers (J Pathol, 1999), Freddie Bray (CA Cancer J Clin, 2018), Sergei I Grivennikov (Cell, 2010), Douglas Hanahan (Cell, 2011), Philip E Castle (Cancer Epidemiol Biomarkers Prev, 2001), Ahmedin Jemal (CA Cancer J Clin, 2011), Harald zur Hausen (Nat Rev Cancer, 2002), and “Papillomaviruses and cancer: from basic studies to clinical application’ (Nat Rev Cancer, 2002) is the minimum with 35 citations (Table S6, http://links.lww.com/MD/L42). Generally, most of the cited references are based on reviews (60%) on inflammation in cervical cancer. In addition, an association between cervical inflammation and high-grade cervical neoplasia in women infected with oncogenic HPV A” (Cancer Epidemiol Biomarkers Prev, 2001), the other 9 cited references in Table S6, http://links.lww.com/MD/L42 are all from Top journals, which also increases the importance of this research field of inflammation in cervical cancer. The top 3 citations of documents are “A Review of the Clinical Side Effects of Bone Morphogenetic Protein-2” (Aaron W James, University of California), “M2 macrophages induced by prostaglandin E2 and IL-6 from cervical carcinoma are switched to activated M1 macrophages by CD4 + Th1 cells” (Moniek Heusinkveld, Leiden University Medical Center), and “CSF1R inhibition delays cervical and mammary tumor growth in murine models by attenuating the turnover of tumor-associated macrophages and enhancing infiltration by CD8 + T cells” (Debbie C Strachan, Novartis Institutes), and “Increased PADI4 expression in blood and tissues of patients with malignant tumors” is the only article by a Chinese team (Xiaotian Chang, Shandong Academy of Medical Sciences) in the top 10 citations of documents (Table S7, http://links.lww.com/MD/L43). Reviews accounted for 4 titles with 1076 citations, and other articles accounted for 6 titles with 1240 citations. As shown in Figure 4B, the top-cited documents in this field are not closely connected, and the article by Heusinkveld presents a certain connection and has guided research work in the later stage.

### 3.7. Keywords analysis

**Figure 5.**
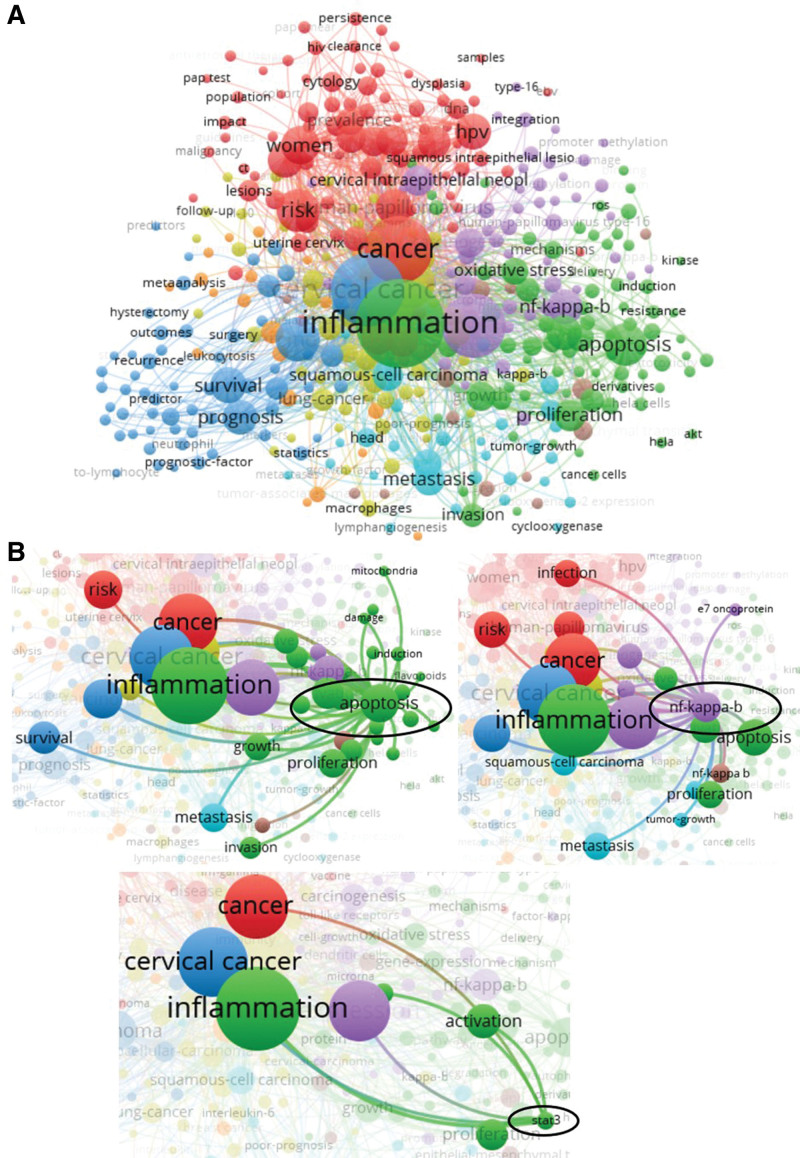
Co-occurrence analysis of keywords. (A) The network visualization of total keywords (398 items) was conducted. (B) The crosstalk of keywords “apoptosis,” “NF-κB” and “STAT3” were presented.

Figure 5A shows the largest keyword subnetwork and its cluster map. Undoubtedly, “inflammation” and “cervical cancer” are the heart of the network and they relate to the keywords of “apoptosis,” “HPV,” “NF-κB,” “oxidative stress,” oxidative stress. There is a close relationship between “apoptosis” and “NF-κB” (Fig. 5B), which also regulates HPV infection, tumor inflammatory response, inhibit tumor cell proliferation, differentiation and metastasis, and increase tumor diagnosis. In addition, STAT3 expression was associated with inflammation and tumor progression in cervical cancer (Fig. 5B). Thus, keyword analysis is beneficial for exploring potential strategies and research directions for cervical cancer diagnosis and treatment via inflammation.

## 4. Discussion

Over the last 2 decades, research on inflammation in cervical cancer has been steadily increasing and has gradually attracted the attention of researchers and organizations. Even though the research institutions from China are active, there is no extensive collaboration and exchange of papers by Chinese scholars. *PLOS One* is a popular journal on inflammation in cervical cancer. Instead, authors from other countries perform better, for example, the Sjoerd H. Van Der Burg is the most widely cited author and “M2 macrophages induced by prostaglandin E2 and IL-6 from cervical carcinoma are switched to activated M1 macrophages by CD4 + Th1 cells” (Moniek Heusinkveld, Leiden University Medical Center) is the most cited article of inflammation in cervical cancer. Keywords associated with “apoptosis,” “HPV,” “NF-κB,” and “oxidative stress have been used in many studies, and there may be more research ideas in the future.

Inflammatory reactions play a decisive role in tumor progression, which also affect the body immune monitoring and response to treatment.^[22]^ In recent years, increasing attention has been paid to tumor immunology because tumors can effectively suppress immune surveillance and the balance of the body immune system against tumors by activating negative regulatory pathways related to immune homeostasis (also known as checkpoints) or by using the inflammatory tumor microenvironment to evade detection.^[23,24]^ The well-known programmed death-ligand 1 (PD-L1)/programmed death 1 (PD-1) axis is an important negative feedback loop that ensures immune homeostasis, and is also an important axis for limiting tumor immunity.^[25]^ Despite the clinical success of antibodies against PD-L1/PD-1, only a small percentage of patients show a durable response, suggesting the need for a broader understanding of cancer immunity and inflammation.^[26,27]^ Defining the basic rules of engagement that control the molecular and cellular mechanisms of tumor-promoting inflammation is crucial for further development of anticancer therapeutics.^[28]^

Targeting inflammation becomes a potential strategy for cancer treatment. Such as, p53 antagonizes the transcription of NF-κB, which is a key positive regulator of inflammation, and loss of functional p53 leads to increased NF-κB signaling in the tumor microenvironment^[29,30]^ and triggers DNA damage-induced inflammatory pathways.^[31,32]^ Additionally, the mechanism by which oncogene activation leads to overproduction of inflammatory cytokines and chemokines (including IL-1α, IL-1β, CCL2, and CXCL1) may be a unifying mechanism for triggering inflammation in many cancers.^[33]^ Some tumor development and progression are initially driven by hepatitis B virus, HCV, or HPV, which promotes different inflammatory responses,^[28]^ including Toll-like receptor 2, TLR4, interferon gene stimulation, cyclic GMP-AMP synthase, and inflammasome sensors.^[34]^ It is clear that persistent infection with high-risk HPV is an independent risk factor for cervical cancer, and that some HPV-infected patients develop chronic inflammation, which eventually leads to cervical cancer.^[9,35]^ However, another emerging paradigm is that many cancers may be promoted by the commensal microbiota, activating the long-range release of microbial metabolites through inflammation.^[36]^ These microorganisms and microbial products can travel with the tumor to the metastatic site and act as a source of inflammation during the metastatic process,^[37]^ and broad-spectrum antibiotic treatment leads to enhanced lung metastasis of LLC and B16-F10 cells by altering the gut microbiome composition in an IL-11-dependent manner.^[38]^ Furthermore, the type of tumor cell death may be important in response to therapeutic and immune mechanisms, as apoptosis and autophagy reduce inflammation. DAMPs released as a result of necrosis or pyroptosis are strong inducers of inflammation, and this inflammation may affect tumor growth mediated by adjacent untransformed cells.^[39]^

In this study, we discovered that inflammation in cervical cancer has recently received considerable attention from researchers in various countries and regions. CD4^+^ Th1 cells from cervical carcinoma stimulate the tumor-rejection environment by converting M2 macrophages into classical proinflammatory M1 macrophages via prostaglandin E2 and IL-6,^[40]^ and the maintaining efficacious tumor-associated macrophage depletion is an effective anticancer therapy for cervical cancer.^[41]^ Cytokines involved in inflammatory processes and various functions have attracted the attention of researchers. IL-1, IL-6, IL-10, IFN-γ, transforming growth factor beta, and other cytokines have been found to have different trends in different degrees of cervical cancer, which has certain predictive significance for tumor progression and prognosis.^[7,9,39]^ Inflammatory factors are important markers for disruption of the cervical epithelial barrier. The cervical epithelial barrier retains the appropriate microbiota to prevent the entry of pathogenic bacteria and viruses into the cervical stroma, which affects the epithelium through the production of metabolites and secreted factors, and induces inflammatory responses and disruption of the epithelial barrier.^[42]^ Cervical antigen-presenting cells sense microbial lipopolysaccharide and activate immune responses through TLR-4 signaling and NF-κB pathways, leading to the secretion of chemokines and cytokines that initiate inflammation.^[43]^ Women with higher high-sensitivity C-reactive protein levels have lower rates of spontaneous cervical cancer degeneration, supporting the role of host inflammatory status in cervical cancer development, suggesting that high-sensitivity C-reactive protein levels help monitor low-grade squamous intraepithelial lesions.^[44]^

Further keyword analysis showed that STAT3 and NF-κB are associated with cell death, inflammation, and tumor progression in cervical cancer. HPV E5/E6/E7 oncogenes activate mitogen-activated protein kinases, hypoxia-inducible factor-1α, PI3K/AKT, STAT3/NF-κB, and microRNA among other signaling pathways that regulate the PD-L1/PD-1 axis to promote HPV-induced cervical carcinogenesis.^[45]^ During the initial phase of HPV infection, NF-κB activity is triggered as a part of the normal host innate immune response; however, during cancer initiation, HPV downregulates NF-κB to liquidate the inhibitory activity for its replication triggered by the innate and adaptive immune response leading to persistent HPV infection, and during the progression to high-grade intraepithelial neoplasia and cervical cancer, NF-κB becomes constitutionally activated again.^[46–48]^ Autocrine STAT3 activation in HPV-positive cervical cancer is mediated by IL-6, and activation of the transcription factor NF-κB is required for IL-6 production and subsequent STAT3 activation,^[49]^ and piperine enhances mitomycin-C-induced cell death in human cervical cancer by blocking the p-STAT3/NF-κB p65 and Bcl-2 signaling pathways.^[50]^ The supernatants of cervical-derived carcinoma cell lines HeLa, SiHa, and C-33A downregulated the activation of transcription factors characteristic of M1 macrophages (STAT1 and NF-κB-p65) and induced MCP-1, IL-6, IL-8, IL-10, G-CSF, GM-CSF, PDGF-AA, PDGF-BB, and vascular endothelial growth factor secretion and response to the microenvironment, which favors tumor growth.^[51]^ FI16 activates interferon gene stimulation-TBK1-mediated immunoregulation and subsequently activates the downstream NF-κB pathway, which interacts with the proximal region of the PD-L1 promoter to facilitate PD-L1 expression, thus promoting cervical cancer progression.^[52]^ In addition, the relationship between STAT3 and NF-κB and tumor progression has been directly confirmed in cervical cancer cells. The NF-κB signaling pathway mediates cell death, growth, migration, invasion, and drug-sensitivity,^[53–58]^ and NF-κB p65 O-GlcNAcylation promotes lung metastasis of cervical cancer cells by activating CXCR4 expression.^[54]^ Lycopene sensitizes the cervical cancer HeLa cells to cisplatin via targeting NF-κB pathway.^[53]^ STAT3/IRF1 pathway activation sensitizes cervical cancer cells to chemotherapeutic drugs; pTyr705-STAT3 correlates with nuclear IRF1 expression in cervical cancer, and high IRF1 expression in pretreatment cervical cancer biopsy cells is associated with a significantly better response to neoadjuvant radio/chemotherapy.^[57]^ The cytokine IL-6 cascades with the transcription factors STAT3 and NF-κB and promotes the occurrence and development of cervical cancer,^[49,59]^ which can provide a new method for further searching for a more accurate new marker that can reflect disease changes and evaluate prognosis, as well as immunotherapy in the future, and it is great significance to study related mechanisms and develop targeted drugs of IL-6, STAT3, and NF-κB.

## 5. Limitations

This study had some limitations. First, the deadline for research publications is December 31, 2022, but WOSCC will continue to update, and many documents are still being updated by 2022. Besides, the terms of “cervical cancer,” “cervix cancer,” “cervix neoplasm,” “cervical neoplasm,” “inflammation,” “inflammatory response,” “inflammatory cytokine,” “English,” “Article” and “Revies” were selected to define the topic of the studies, not all documents were completely obtained, such as the Meeting, Case Report, Clinical Trial, Patent and other multiple document types. Third, because the search was limited to the WOS Core Collection databases, some documents, such as MEDLINE®, the KCI-Korean Journal Database, and SciELO Citation Index, were missed. However, we believe that the overall situation and general trends of these analyses are consistent with research blueprints for inflammation in cervical cancer.

## 6. Conclusion

This study analyzed the research conditions of inflammation in cervical cancer using a bibliometric analysis. The data showed that inflammation in cervical cancer might be an interesting field of research; however, it has an unimpressive number of publications and low-frequency cooperation. IL-6-, STAT3-, and NF-κB-mediated cell death, inflammatory response, tumorigenesis, tumor progression, and tumor microenvironment of cervical cancer From the perspective of precision medicine, more substantive research articles can greatly promote scientific value, strengthen the communication and cooperation between research teams and institutions, produce more extensive research results, and greatly promote the clinical diagnosis and treatment of cervical cancer.

## Author contributions

**Conceptualization:** Meili Kang, Jianing Li.

**Data curation:** Meili Kang, Junling Qiu, Hong Wei.

**Formal analysis:** Junling Qiu, Hong Wei.

**Methodology:** Hong Wei.

**Resources:** Hong Wei.

**Supervision:** Jianing Li.

**Visualization:** Meili Kang, Junling Qiu.

**Writing – original draft:** Meili Kang, Hong Wei.

**Writing – review & editing:** Jianing Li.

## Supplementary Material














