# Supplementary material for: A bibliometric analysis of global research trends of inflammation in cervical cancer: A review
Source: Medicine (Baltimore). 2023 Dec 8;102(49):e36598. doi: 10.1097/MD.0000000000036598 (PMC10713142; doi:10.1097/MD.0000000000036598)
Supplement: Supplementary file 1 [file medi-102-e36598-s001.docx]

Table S1. The Top 10 funding sources

| Ranking | Funding Source | Frequency |
| --- | --- | --- |
| 1 | National Natural Science Foundation of China NSFC | 122 |
| 2 | United States Department of Health Human Services | 104 |
| 3 | National Institutes of Health NIH USA | 103 |
| 4 | NIH National Cancer Institute NCI | 68 |
| 5 | NIH National Institute of Allergy Infectious Diseases NIAID | 23 |
| 6 | Conselho Nacional De Desenvolvimento Cientifico E Tecnologico CNPQ | 21 |
| 7 | European Commission | 20 |
| 8 | Consejo Nacional De Ciencia Y Tecnologia Conacyt | 16 |
| 9 | Fundacao De Amparo A Pesquisa Do Estado De Sao Paulo Fapesp | 14 |
| 10 | Ministry Of Education Culture Sports Science And Technology Japan MEXT | 14 |
