# Supplementary material for: A bibliometric analysis of global research trends of inflammation in cervical cancer: A review
Source: Medicine (Baltimore). 2023 Dec 8;102(49):e36598. doi: 10.1097/MD.0000000000036598 (PMC10713142; doi:10.1097/MD.0000000000036598)
Supplement: Supplementary file 2 [file medi-102-e36598-s002.docx]

Table S2 Top 10 countries/regions of inflammation in cervical cancer

| Rank | Country/Region | Documents | Citations | Total link strength | Links |
| --- | --- | --- | --- | --- | --- |
| 1 | China | 366 | 5761 | 65 | 22 |
| 2 | USA | 194 | 6367 | 119 | 42 |
| 3 | India | 81 | 1304 | 40 | 19 |
| 4 | Japan | 53 | 1248 | 13 | 6 |
| 5 | Brazil | 50 | 1062 | 17 | 9 |
| 6 | Germany | 44 | 759 | 37 | 21 |
| 7 | South Korea | 44 | 1176 | 15 | 6 |
| 8 | Italy | 39 | 941 | 26 | 15 |
| 9 | UK | 27 | 619 | 29 | 19 |
| 10 | Iran | 23 | 408 | 9 | 6 |
