# Supplementary material for: A bibliometric analysis of global research trends of inflammation in cervical cancer: A review
Source: Medicine (Baltimore). 2023 Dec 8;102(49):e36598. doi: 10.1097/MD.0000000000036598 (PMC10713142; doi:10.1097/MD.0000000000036598)
Supplement: Supplementary file 3 [file medi-102-e36598-s003.docx]

Table S3 Top 10 the most productive organizations

| Rank | Organizations | Country | Documents | Citations | Total link strength |
| --- | --- | --- | --- | --- | --- |
| 1 | Sichuan University | China | 22 | 504 | 27 |
| 2 | University of California | USA | 18 | 896 | 21 |
| 3 | University of Sao Paulo | Brazil | 17 | 402 | 41 |
| 4 | Fudan University | China | 16 | 219 | 20 |
| 5 | University of Texas | USA | 16 | 260 | 33 |
| 6 | China Medicinal University | China | 12 | 195 | 44 |
| 7 | Sun Yat Sen University | China | 12 | 318 | 12 |
| 8 | Nanjing Medical University | China | 11 | 157 | 17 |
| 9 | Shandong University | China | 11 | 390 | 15 |
| 10 | University of NACL Autonoma Mexico | Mexico | 10 | 77 | 29 |
