# Supplementary material for: A bibliometric analysis of global research trends of inflammation in cervical cancer: A review
Source: Medicine (Baltimore). 2023 Dec 8;102(49):e36598. doi: 10.1097/MD.0000000000036598 (PMC10713142; doi:10.1097/MD.0000000000036598)
Supplement: Supplementary file 4 [file medi-102-e36598-s004.docx]

Table S4 Top 10 journals with the largest number of publications

| Rank | Journals | Documents | 2021 Impact Factor | 2021 JCR Partition |
| --- | --- | --- | --- | --- |
| 1 | PLOS One | 26 | 3.752 | Q2 |
| 2 | Oncology Letters | 23 | 3.111 | Q3 |
| 3 | International Journal of Gynecological Cancer | 19 | 4.661 | Q1 |
| 4 | Oncotarget | 15 | / | / |
| 5 | Scientific Reports | 15 | 4.996 | Q2 |
| 6 | International Journal of Clinical and Experimental Pathology | 14 | / | / |
| 7 | Oncology Reports | 14 | 4.136 | Q3 |
| 8 | Asian Pacific Journal of Cancer Prevention | 12 | / | / |
| 9 | Mediators of Inflammation | 12 | 4.529 | Q3 |
| 10 | Gynecologic Oncology | 11 | 5.304 | Q1 |
