# Supplementary material for: A bibliometric analysis of global research trends of inflammation in cervical cancer: A review
Source: Medicine (Baltimore). 2023 Dec 8;102(49):e36598. doi: 10.1097/MD.0000000000036598 (PMC10713142; doi:10.1097/MD.0000000000036598)
Supplement: Supplementary file 5 [file medi-102-e36598-s005.docx]

Table S5 Top 10 active authors with most documents

| Authors | Organizations | Country | Documents | Citations | h-index |
| --- | --- | --- | --- | --- | --- |
| Luisa L. Villa | Universidade de São Paulo | Brazil | 11 | 331 | 37 |
| Enrique Boccardo | Universidade de São Paulo | Brazil | 7 | 208 | 13 |
| Ana P. Lepique | Universidade de São Paulo | Brazil | 6 | 193 | 13 |
| Sjoerd H. Van Der Burg | Leiden University Medical Center, | Netherlands | 6 | 626 | 4 |
| Yu Zhang | Harbin Medical University | China | 6 | 129 | 2 |
| Christoph Grimm | Christoph Grimm | Austria | 5 | 139 | 20 |
| Stephan Polterauer | Christoph Grimm | Austria | 5 | 139 | 20 |
| Alexander Reinthaller | Christoph Grimm | Austria | 5 | 139 | 20 |
| Melissa M. Herbst-kralovetz | University of Arizona | USA | 5 | 261 | 16 |
| Pawel Laniewski | University of Arizona | USA | 5 | 261 | 16 |
