# Supplementary material for: A bibliometric analysis of global research trends of inflammation in cervical cancer: A review
Source: Medicine (Baltimore). 2023 Dec 8;102(49):e36598. doi: 10.1097/MD.0000000000036598 (PMC10713142; doi:10.1097/MD.0000000000036598)
Supplement: Supplementary file 6 [file medi-102-e36598-s006.docx]

Table S6 Top 10 Co-citation of cited reference of inflammation in cervical cancer

| \| Rank \| Title \| First author \| Source \| Type \| Publication year \| Total citations \| \| --- \| --- \| --- \| --- \| --- \| --- \| --- \| \| 1 \| Inflammation and cancer \| Lisa M Coussens \| Nature \| Review \| 2002 \| 88 \| \| 2 \| Inflammation and cancer: back to Virchow? \| Fran Balkwill \| Lancet \| Review \| 2001 \| 64 \| \| 3 \| Cancer-related inflammation \| Alberto Mantovani \| Nature \| Review \| 2008 \| 63 \| \| 4 \| Human papillomavirus is a necessary cause of invasive cervical cancer worldwide \| Jan MM Walboomers \| J Pathol \| Article \| 1999 \| 63 \| \| 5 \| Global cancer statistics 2018: GLOBOCAN estimates of incidence and mortality worldwide for 36 cancers in 185 countries \| Freddie Bray \| CA Cancer J Clin \| Article \| 2018 \| 59 \| \| 6 \| Immunity, inflammation, and cancer \| Sergei I Grivennikov \| Cell \| Review \| 2010 \| 55 \| \| 7 \| Hallmarks of cancer: the next generation \| Douglas Hanahan \| Cell \| Review \| 2011 \| 48 \| \| 8 \| An association of cervical inflammation with high-grade cervical neoplasia in women infected with oncogenic human papillomavirus (HPV) \| Philip E Castle \| Cancer Epidemiol Biomarkers Prev \| Article \| 2001 \| 41 \| \| 9 \| Global cancer statistics \| Ahmedin Jemal \| CA Cancer J Clin \| Article \| 2011 \| 38 \| \| 10 \| Papillomaviruses and cancer: from basic studies to clinical application \| Harald zur Hausen \| Nat Rev Cancer \| Review \| 2002 \| 35 \| |
| --- | --- | --- | --- | --- | --- | --- | --- | --- | --- | --- | --- | --- | --- | --- | --- | --- | --- | --- | --- | --- | --- | --- | --- | --- | --- | --- | --- | --- | --- | --- | --- | --- | --- | --- | --- | --- | --- | --- | --- | --- | --- | --- | --- | --- | --- | --- | --- | --- | --- | --- | --- | --- | --- | --- | --- | --- | --- | --- | --- | --- | --- | --- | --- | --- | --- | --- | --- | --- | --- | --- | --- | --- | --- | --- | --- | --- | --- |
