# Supplementary material for: A bibliometric analysis of global research trends of inflammation in cervical cancer: A review
Source: Medicine (Baltimore). 2023 Dec 8;102(49):e36598. doi: 10.1097/MD.0000000000036598 (PMC10713142; doi:10.1097/MD.0000000000036598)
Supplement: Supplementary file 7 [file medi-102-e36598-s007.docx]

Table S7 Top 10 citation analysis of documents of inflammation in cervical cancer

| Rank | Title | First author | Source | Type | Publication year | Total citations |
| --- | --- | --- | --- | --- | --- | --- |
| 1 | A Review of the Clinical Side Effects of Bone Morphogenetic Protein-2 | Aaron W James | Tissue Eng Part B Rev | Review | 2016 | 458 |
| 2 | M2 macrophages induced by prostaglandin E2 and IL-6 from cervical carcinoma are switched to activated M1 macrophages by CD4+ Th1 cells | Moniek Heusinkveld | J Immunol | Article | 2011 | 268 |
| 3 | CSF1R inhibition delays cervical and mammary tumor growth in murine models by attenuating the turnover of tumor-associated macrophages and enhancing infiltration by CD8 + T cells | Debbie C Strachan | Oncoimmunology | Article | 2013 | 240 |
| 4 | Anticancer effects of the microbiome and its products | Laurence Zitvogel | Nat Rev Microbiol | Review | 2017 | 227 |
| 5 | Inflammation and cancer | Mariko Murata | Environ Health Prev Med | Review | 2018 | 210 |
| 6 | Toxicological effects of inorganic nanoparticles on human lung cancer A549 cells | Soo-Jin Choi | J Inorg Biochem | Article | 2009 | 200 |
| 7 | Cervical intraepithelial neoplasia disease progression is associated with increased vaginal microbiome diversity. | Anita Mitra | Sci Rep | Article | 2015 | 195 |
| 8 | Aldose reductase enzyme and its implication to major health problems of the 21(st) century | Polyxeni Alexiou | Curr Med Chem | Review | 2009 | 181 |
| 9 | Identification of known drugs that act as inhibitors of NF-kappaB signaling and their mechanism of action | Susanne C Miller | Biochem Pharmacol | Article | 2010 | 179 |
| 10 | Increased PADI4 expression in blood and tissues of patients with malignant tumors | Xiaotian Chang | BMC Cancer | Article | 2009 | 158 |
